# Application of Micro-Engineered Kidney, Liver, and Respiratory System Models to Accelerate Preclinical Drug Testing and Development

**DOI:** 10.3390/bioengineering9040150

**Published:** 2022-04-02

**Authors:** Hanieh Gholizadeh, Shaokoon Cheng, Agisilaos Kourmatzis, Hanwen Xing, Daniela Traini, Paul M. Young, Hui Xin Ong

**Affiliations:** 1 Macquarie Medical School, Faculty of Medicine, Health, and Human Sciences, Macquarie University, Ryde, NSW 2109, Australia; hanieh.mohammad-gholizadeh-@hdr.mq.edu.au (H.G.); daniela.traini@mq.edu.au (D.T.); 2 Respiratory Technology, The Woolcock Institute of Medical Research, The University of Sydney, Sydney, NSW 2037, Australia; p.young@mq.edu.au; 3 School of Engineering, Faculty of Science and Engineering, Macquarie University, Ryde, NSW 2113, Australia; shaokoon.cheng@mq.edu.au; 4 School of Aerospace, Mechanical and Mechatronic Engineering, The University of Sydney, Sydney, NSW 2006, Australia; agisilaos.kourmatzis@sydney.edu.au; 5 Faculty of Engineering and Information Technology, University of Technology Sydney, Sydney, NSW 2007, Australia; xinghanwen9@gmail.com; 6 Department of Marketing, Macquarie Business School, Macquarie University, Ryde, NSW 2109, Australia

**Keywords:** organ-on-chip, metabolism, toxicology, drug transport, body-on-chip, disease-on-chip, drug discovery

## Abstract

Developing novel drug formulations and progressing them to the clinical environment relies on preclinical in vitro studies and animal tests to evaluate efficacy and toxicity. However, these current techniques have failed to accurately predict the clinical success of new therapies with a high degree of certainty. The main reason for this failure is that conventional in vitro tissue models lack numerous physiological characteristics of human organs, such as biomechanical forces and biofluid flow. Moreover, animal models often fail to recapitulate the physiology, anatomy, and mechanisms of disease development in human. These shortfalls often lead to failure in drug development, with substantial time and money spent. To tackle this issue, organ-on-chip technology offers realistic in vitro human organ models that mimic the physiology of tissues, including biomechanical forces, stress, strain, cellular heterogeneity, and the interaction between multiple tissues and their simultaneous responses to a therapy. For the latter, complex networks of multiple-organ models are constructed together, known as multiple-organs-on-chip. Numerous studies have demonstrated successful application of organ-on-chips for drug testing, with results comparable to clinical outcomes. This review will summarize and critically evaluate these studies, with a focus on kidney, liver, and respiratory system-on-chip models, and will discuss their progress in their application as a preclinical drug-testing platform to determine in vitro drug toxicology, metabolism, and transport. Further, the advances in the design of these models for improving preclinical drug testing as well as the opportunities for future work will be discussed.

## 1. Introduction

Intensive and costly preclinical tests on novel therapeutic agents often involve animal studies prior to human trials. In addition to being ethically controversial, animal models have been increasingly criticized for their limited ability to predict the efficacy, safety, and toxicity of numerous drugs in humans [1,2]. Animal testing is predisposed—by its xenogeneic nature and failure to represent the complicated anatomical and physiological systems in humans—to lead to discordant results and, consequently, to failures in translating the results to clinical trials. Conventional in vitro models that utilize two-dimensional (2D) or three-dimensional (3D) cell cultures, on the other hand, are limited in their predictive capabilities of in vivo conditions due to the absence of critical physiological factors, such as fluid flow and biomechanical forces [3]. Consequently, the development of novel methodologies that could accurately represent the physiological conditions of the human body in an in vitro platform to perform drug testing is of critical importance to accelerate the development of novel therapeutics that can be used in clinical settings.

The development of micro-structured functional human organ models, known as organ-on-chip technology, is a potential solution for providing a physiologically relevant in vitro platform and has attracted increasing interest [3]. Organ-on-chips aim to provide a translational model for human organs to predict human responses to therapeutic agents. As a result, they can deliver reliable and accurate outcome measures and also likely have the potential to predict clinical trial results. Since its advent, organ-on-chip technology has been widely studied and found to offer numerous advantages in recapitulating organ physiology to study human diseases and its potential for drug testing. Current reviews on the application of organ-on-chips for in vitro drug tests mainly focus on models of certain organs, their microfluidic designs and structures, and their relevance to healthy or diseased tissues whilst providing examples of their potential application for testing drugs [4,5,6,7,8]. In addition, these reviews elaborate on the advances in organ-on-chips and their future opportunities for commercialization of these platforms for drug testing [3,9,10,11]. Table 1 provides an overview of the different relevant reviews and their comparison with the current review on the application of organ-on-chips for drug testing. This review will provide a pharmaceutical perspective by focusing on the application of organ-on-chips for different preclinical drug tests with improved relevance. Hence, the studies on organ-on-chips will be discussed based on the assays used to evaluate drug efficacy and toxicity in vitro, which are important for drug development and commercialization. Further, the advantages of organ-on-chips in drug testing will be elaborated based on their physiologically relevant dynamic design [12,13,14], emulation of the inter-organ crosstalk in the human body [15,16], enabling patient-specific drug testing [17], and high throughput time-efficient assays by integrated miniaturised analytical tools [18,19]. Liver, kidney, and respiratory system models will be the focus of this review due to their broad applicability in toxicology [20], drug metabolism [21], and drug delivery/transport [22] studies.

## 2. Drug Testing Capabilities Using Liver-, Kidney-, and Lung-on-Chip Models

**Figure 1:**
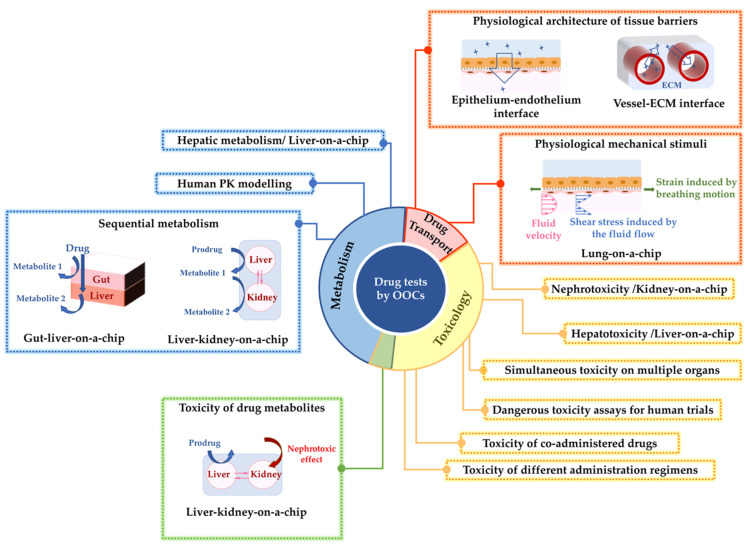
A summary of the investigations on three important drug assessments: drug metabolism, toxicology, and drug transport using liver-, kidney-, and lung-on-chip models. The figure summarizes the advantages of these organ-on-chip models to enhance drug testing.

Liver-, kidney-, and lung-on-chip models have recreated some key functions of their respective organs for drug metabolism, expression of cytotoxic response to drugs, and barrier function against drug permeation. Figure 1 summarizes how these organ-on-chips have been used for studies on drug metabolism, toxicology, and transport. 

### 2.1. Drug Metabolism Studies

The principal, although not the sole, site for metabolism of nearly all ingested drugs is the liver [23]. Consequently, first-pass hepatic metabolism has a paramount influence on the efficacy and side effects of drugs, which highlights the importance of simulating this biological process during drug testing. Organ-on-chip technology has been utilized for screening drug metabolism, where recapitulation conditions of the liver tissue have been the main focus [24,25]. Table 2 summarizes the studies on the metabolism of different drugs using liver-on-chip models and the interconnection of the liver with other organs-on-chip.

The utilization of organ-on-chip technology to construct the network of multiple organs is beneficial to mimic the complex interaction between organs in metabolizing drugs, prodrugs, and micronutrients in vivo. For instance, a liver–kidney-on-chip with interconnected chambers for liver and kidney cell culture was developed by Tehobald et al. [26] to imitate sequential hepatic and renal metabolism of vitamin D. The device could mimic hepatic metabolism of vitamin D to 25-hydroxyvitamin D and its further metabolism to 1,25-dihydroxyvitamin D by the kidney analog. The latter bioactive metabolite is known for its anti-tumor effect and for inducing differentiation in multiple tumor cell types, such as acute myeloid leukemia cells.

Studying the drug metabolism process in multi-organs-on-chip models and their comparison with single-organ-on-chip models can shed insights into understanding the tissue–tissue crosstalk and individual contribution to the metabolic process [27]. In another study, Choe et al. reproduced the first-pass-metabolism process in a microfluidic gut–liver chip with two chambers separated by a membrane to culture gut epithelial and liver cells [28]. The gut cells’ culture chamber was located on top of the liver cells’ chamber, so that the drug molecules passing across the gut epithelial barrier could reach the liver cells. This enables emulation of the simultaneous drug absorption in the gut and metabolic reaction in the liver that the drug goes through after oral intake. The metabolic activity of the gut–liver chip was compared to the gut monoculture system, and the gut epithelial cells were highlighted to have significantly higher contribution in the metabolism of apigenin.

Another important factor that needs to be considered when designing in vitro drug-metabolism models is that the rates of absorption and metabolism are influenced by the volumes and dimensions of the designed organ model [29]. Hence, it is important to replicate the relative sizes of organ analogs and the circulating fluid flow connecting them based on the physiology of the human body in order to achieve accurate pharmacokinetic (PK) modelling using multi-organs-on-chip. Some studies on the application of multi-organs-on-chips to replicate human drug metabolism have focused on the design of devices based on mathematically modelled PK profiles [30,31]. These microfluidic chips are promising tools to emulate human-relevant PK in vitro. For instance, the first-pass metabolism of orally taken paracetamol was replicated in a gut–liver-on-chip [31]. The design parameters of the chip were such that the surface area of the gut and the volume of the liver compartment was optimized based on a mathematical PK model close to the human PK.

Taken together, organ-on-chips have been able to successfully model the metabolism of numerous drugs, either by a single tissue or multiple tissues interconnected. As shown in Table 2, the majority of these devices are evaluated based on the testing of only one drug molecule. Future research is needed to test a wide variety of drug candidates and formulations, with their excipients using these devices to validate them against existing in vivo observations and to study any drug–drug interactions that may occur as a result of concomitant drug uptake.

**Table 2:** Summary of the drug metabolism and drug toxicity studies that include liver and/or kidney tissue models on organ-on-chips (OOCs) and multi-organ-on-chips (MOCs).

Drug	Toxicology	Metabolism	Tissue(s)	Reference
diclofenac acetaminophen	✓		liver	[18]
troglitazone	✓		liver	[19]
acetaminophen	✓		liver	[32,33,34,35,36]
acetaminophen	✓	✓	liver	[37,38]
acetaminophen isoniazid rifampicin	✓	✓	liver	[39]
rifampin ketoconzazole acetaminophen	✓	✓	liver	[40]
bupropion tolbutamide omeprazole testosterone		✓	liver	[24]
7-ethoxy-4-trifluoromethyl coumarin		✓	liver	[25]
acetaminophen chlorpromazine tacrine	✓		liver	[41]
ccetaminophen fialuridine	✓	✓	liver	[42]
diclofenac	✓	✓	liver	[43]
cadmium aspirin caffein troglitazone rosiglitazone pioglitazone acetaminophen	✓		liver	[2]
cisplatin	✓		kidney	[14]
adriamycin	✓		kidney	[44]
gentamicin	✓		kidney	[45]
polymyxin B	✓		kidney	[46]
carboxylated polystyrene nanoparticles	✓		GI tract–liver	[47]
troglitazone	✓	✓	liver–intestine liver–skin	[48]
apigenin		✓	gut–liver	[28]
epirubicine irinotecan cyclophosphamide		✓	small intestine–liver–lung	[29]
ifosfamide verapamil	✓	✓	liver–kidney	[49]
paracetamol		✓	liver–gut	[31]
mannitol propranolol caffeine		✓	GI–liver	[50]
combination of genistein and dacarbazine		✓	intestine–liver	[51]
5-fluorouracil	✓		liver–tumor–marrow	[52]
paracetamol	✓	✓	liver–kidney	[53]
diclofenac ketoconazole hydrocortisone acetaminophen	✓		liver–heart–skin	[54]
luteolin		✓	liver–tumor	[30]
capecitabine tegafur		✓	liver–cancer intestine–liver–cancer–connective tissue	[27]
digoxin	✓		intestine–kidney	[55]
ifosfamide	✓	✓	liver–kidney	[56]
vitamin D		✓	liver–kidney	[26]

### 2.2. Toxicology

Drug-induced toxicity is one of the major reasons for the failure of drug candidates and the withdrawal of approved drugs from the market [57]. The main reason for drug toxicity is the undesirable off-target activity of drug molecules or their reactive metabolites. Determining toxicity early in the drug discovery pipeline remains a challenge, as animal studies do not efficiently predict toxicity responses in human [3,58]. Therefore, accurate high-throughput assays for toxicity prediction are highly valuable in the pharmaceutical industry to reject potentially toxic drug candidates at the early stages. In this regard, the application of new technologies that could enable toxicity studies to be done in the context of organotypic biology as highly predictive models has attracted significant interest [57,59]. Table 2 summarizes the studies utilizing kidney and liver tissues-on-chip for drug toxicity assays (since nephrotoxicity [14,44,45,46,49,55,56] and hepatotoxicity [32,33,38,40,41,42,43,51,60] are the most common adverse effects reported in drug development).

#### 2.2.1. Toxicology Studies by the Kidney- or Liver-on-Chip Models

The liver- and kidney-on-chip models were developed to have in vivo-like tissue functionalities, and they have demonstrated superiority over the conventional cell culture platforms in predicting toxic drug responses [14]. For instance, a kidney-glomerulus-on-chip was developed using pluripotent stem cells differentiated into kidney podocytes [44]. The cells in this device expressed morphological, molecular, and functional characteristics similar to mature human podocytes. Indeed, the renal toxicity associated with albuminuria induced by a cancer drug, Adriamycin, was successfully simulated by this device. Furthermore, the hepatotoxic effect of acetaminophen was also modelled by liver-on-chip models, where the toxic response of the tissue model to the drug treatment was evaluated based on the disruption in basic liver function or secretion of biomarkers similar to in vivo observations [34,35].

The application of organ-on-chips in toxicology has also been expanded to simulate specific drug administration schedules. Previous studies have shown that organ-on-chips can differentiate dosage regimens, where the modality of drug administration or the interaction of drugs may influence the toxicity response of organ tissues. A kidney-on-chip model simulated the nephrotoxicity of gentamicin in two different administration regimens: bolus injection and continuous infusion [45]. These different treatment regimens, given in the same dose, led to significantly different nephrotoxicity outcomes, with increased cytotoxicity detected for continuous infusion. Organ-on-chips also enabled the simulation of toxicity response to drug–drug interactions in patients undergoing treatment of multiple diseases with coadministration of medicines. A study by Ma et al. reported an in vitro, 3D, liver-lobule-like microtissue [39] that was used to simulate adverse drug reactions caused by the interaction of acetaminophen and omeprazole, rifampicin, ciprofloxacin, or probenecid. The consequent hepatotoxic effects reported in vivo could be simulated in vitro by this chip. It was observed that the pretreatment of the chip with omeprazole or probenecid resulted in an increased hepatotoxic effect of acetaminophen. However, the toxicity effect was alleviated by pretreatment of the cells with rifampicin or ciprofloxacin.

Another benefit of performing toxicology studies in organ-on-chips is that they can be used to assess poisonous molecules that are not ethically acceptable to subject healthy humans to. One example of this application are studies on radiation-induced injuries and testing the efficacy of radioprotective drugs or assessing radiotherapies [61]. Since the experimental exposure of healthy people to radiation is unethical, the organ-on-chips can offer valuable input in this area to conduct these assessments safely and rapidly. Another study that supports this application is the coculture of mammary epithelial cells and hepatic carcinoma cells on a chip [62]. The chip was exposed to gamma radiation to mimic the space-like environment, where the radioprotection effect of amifostine prodrug on human mammary epithelial cells after metabolism by the liver could be modelled in vitro.

#### 2.2.2. Toxicology Studies by the Kidney- and Liver-on-Chip Models Interconnected with Other Organs

Simulating a network of organs based on physiological PK has been promising in toxicology studies [63]. An example of this network is a lung–liver-on-chip that simulated the decreased toxicity of inhaled toxicants in the lung tissue because of the detoxification process in the liver tissue [64]. Such multi-organs-on-chip platforms also enable the simulation of drug metabolism in one organ and the consequent toxic effect of the formed metabolites in another organ [47,53,55]. Similarly, severe nephrotoxicity of ifosfamide anticancer prodrug observed in the clinic could be emulated by a liver–kidney-on-chip that mimicked the interaction of kidney and liver [56]. Multi-organs-on-chips also allow simultaneous evaluation of the cytotoxic effects on the targeted and untargeted organs, where a study by Theobald et al. was able to model both the hepatotoxicity and nephrotoxicity of Aflatoxin B1 in a kidney–liver-on-chip [65].

The multi-organ-on-chips—in their advanced form involving more organ analogs—could eventually mimic the drug toxicity in the whole human body, significantly helping the prediction of side effects of drug candidates at a very early stage in vitro.

### 2.3. Drug Delivery/Transport

Transport of therapeutics across tissue barriers is one of the major challenges in drug delivery that can influence the bioavailability of drugs [66] and, hence, requires considerations and optimizations during drug discovery. Skin, epithelium, intestine, and blood-brain barrier (BBB) are examples of tissue in the human body that provide effective barriers against the delivery of therapeutics via transdermal, respiratory, oral, and intravenous drug delivery routes, respectively. Drug transport across in vitro tissue barrier models is another assessment where the application of the organ-on-chip models can be beneficial.

#### 2.3.1. Simulation of In Vivo-Level Barrier Functions On-Chip

Organ-on-chips can model tissue-specific barrier functions with reliable physiological relevance to study drug permeation. The organ-on-chip models used for drug permeation studies are mainly designed as dual-chamber structures with a donor and an acceptor chamber. The donor chamber is mainly located on top of the acceptor chamber, and the tissue barrier is represented by the culture of cells on a permeable membrane that separates the two chambers. The quantity of drug transported from the donor to the acceptor chamber is measured to evaluate the efficient permeation of the developed drugs across the tissue barriers [12,50,54]. A study by Chen et al. modelled gastrointestinal (GI) barrier functionality in a GI–liver system [50]. This coculture cell model was able to express the desired physiological relevance, as assessed by the transepithelial electrical resistance (TEER) similar to the human native gut, and allowed emulation of the in vivo absorption of drugs across the gut wall. TEER measures the electrical resistance of the developed epithelium model and is a quantitative representation of the tissue barrier function and formation of tight junctions (TJs) between the cells [67]. Additionally, the GI barrier functionality was demonstrated in terms of the permeability of mannitol, propranolol, and caffeine across the primary intestinal monolayer in this chip. Organ-on-chips can also recreate heterogenous cellular structures, where the transport of substances occurs across adjacent tissues. The goal is to simulate the neighbouring tissues-on-chip with the coculture of different cell types to create structures similar to the native organs in the human body. This was proven feasible by a lung-on-chip developed by Huh et al. that could mimic the interaction between the pulmonary alveoli and the neighboring vascular endothelium in the human lung [12]. Therefore, this model enables the consideration of regulatory functions of both the epithelial and endothelial tissues as a more reliable barrier model for pulmonary drug-delivery tests.

#### 2.3.2. Simulation of Multiple Drug-Delivery Routes On-Chip

Multi-organs-on-chip structured by the coculture of different organs also enabled the evaluation of different drug delivery routes and resultant transport of drug to the target tissue. This was demonstrated in a study by de Melo et al., where the skin barrier and dermal drug absorption were modelled by a Strat-M^®^ membrane incorporated in a heart–liver-body-on-chip system [54]. The chip system mimicked both transdermal and systemic drug delivery routes and, hence, could predict both hepatotoxicity and cardiotoxicity of four model drugs administered to the chip: diclofenac, ketoconazole, hydrocortisone, and acetaminophen. In another study, Ozkan et al. conducted drug permeation studies using a two-vessel structure-on-chip mimicking the vascularized microenvironments of the liver and breast tumors [68]. The two vessels were surrounded by collagen-based extracellular matrix (ECM) with breast cancer cells or liver cells cultured in each vessel. This chip allowed evaluation of particle diffusion from a vessel into its surrounding ECM and back into the vessel, as well as transportation between the two vessels. For this purpose, particles of different sizes were perfused through these vascularized microenvironments to replicate chemotherapy drugs and drugs conjugated with nanoparticles. The resultant permeability of the tumor microenvironment and the accumulation of particles observed in ECM from this chip were consistent with in vivo findings [68,69,70,71,72].

#### 2.3.3. Drug Delivery Tests under In Vivo-Inspired Dynamic Conditions On-Chip

Another added benefit of organ-on-chips is the capability to perform drug transport studies under mechanically dynamic conditions. The physiological mechanical cues in native organs could be emulated in the engineered design of organ-on-chips. This can enable drug-transport studies across tissue barriers under more physiologically realistic conditions. This feature was prominent and was highlighted in the lung-on-chip model that had capabilities to emulate both the strain exerted on the alveolar–capillary barriers by breathing motions and the shear stress on the capillary endothelium induced by the blood flow [12]. The lung-on-chip could mimic the physiological breathing motion via cyclic vacuuming of two channels on the sides. This resulted in cyclic stretching motions of a membrane inside the cell-culture channel, inducing physiological levels of cyclic strain. This dynamic microenvironment was eventually found to influence the permeability of the alveolar–endothelium interface. Higher permeability of nanoparticles across the alveolar–capillary interface was observed when the physiological strain was emulated compared to the device at static condition. The control, static, Transwell culture was prepared with the coculture of the alveolar epithelial cells and capillary endothelial cells on the opposing sides of the membrane. In another study, a human nasal epithelial mucosa-on-chip with a dual-channel structure was developed to model the nasal epithelial barrier against nasal drug delivery [73]. The transport of ibuprofen across the modelled epithelium was evaluated under physiologically relevant flowing fluid conditions in the donor channel. It was observed that the circulating pulsatile fluid flow in the bottom channel resulted in an increase in the drug transport rate compared to the static condition in the chip. This observation was explained by the increase in the convective mass transfer coefficient in the fluid due to the flow condition, which highlights the necessity of conducting nasal drug transport studies under dynamic conditions.

#### 2.3.4. Further Improvements of Drug Delivery Studies by Organ-on-Chips

There remain unexplored areas to improve the relevance of drug-delivery studies by organ-on-chips. For instance, the simulation of realistic drug administration forms, namely the topical delivery of aerosolized solutions or dry powders within the dynamic microenvironments of organ-on-chips, has not been studied. Such simulations enable mimicking of the realistic deposition of fluidized particles to the target tissue and the aerosol characteristics that can influence the efficacy of aerosol drug delivery. These characteristics include particle size and flowrate of aerosolized particles, which can affect the delivered dose [74,75]. Additionally, physiological barriers that adversely affect the efficiency of drug-delivery routes have not been comprehensively studied by organ-on-chips. As a case-in-point, the mucociliary clearance mechanism that limits the residential time of drug particles in human airways is an unexplored area by in vitro models of lower [12,13,76] and upper [73] airways-on-chip. Simulation of this airway defense mechanism by future lung and nasal epithelium-on-chip models can profoundly enhance their physiological relevance and, eventually, their ability to model relevant clinical drug test results in vitro.

## 3. Advantages of Organ-on-Chips for Drug Testing

The benefits of conducting drug tests by organ-on-chip models can be summarized broadly into three main categories. Firstly, organ-on-chips are engineered to better mimic the in vivo physiology and pathophysiology of human organs. In doing so, organ-on-chips support the coculture of cells in one platform, simulation of the mechanical cues, and mimicking human diseased tissues. Secondly, organ-on-chips can facilitate and accelerate the drug-testing procedure by offering in situ sensing tools in their designs. Thirdly, organ-on-chips cultured with patient-derived cells can enhance personalized medicine.

### 3.1. Organ-on-Chips Offer Engineered In Vivo-Inspired Microenvironments

The main advantage of organ-on-chips is their engineered structure, which enables mimicking the microenvironments and functionalities of human organs with the in vivo-inspired architecture, multicellular nature, and biomechanical stimuli that may interfere with drug delivery, absorption, metabolism, and cytotoxic responses.

#### 3.1.1. Cellular Coculture and Organ–Organ Crosstalk

Coculture of multiple cell types in organ-on-chips enables the understanding of the inter-organ crosstalk contribution in drug testing [30]. The interface of epithelial tissue and the neighbouring microvascular endothelium is the most commonly reproduced tissue–tissue interface structure in various organ-on-chips to evaluate drug efficacy [12,76,77,78]. The importance of recapitulating such interfaces lies in their role in regulating the transport of drugs, immune cells, and nutrients that could influence therapeutic outcomes [12]. Importantly, multiple tissue analogs interconnected on-chip with dynamic fluidic channels based on a physiologically correct scale, order, and cell-to-liquid ratio can reproduce the interactions between multiple organs in a whole body [16]. Hence, these devices are expected to simulate the uptake and circulation of therapeutics, the complex process of drug metabolism, the potential toxic or therapeutic effect of one organ’s metabolites on the second organ, and the combined responses of several tissues to drugs and toxicants [30,47,48,49,55]. Such multi-organs-on-chip platforms—designed and scaled based on mathematical pharmacokinetic–pharmacodynamic (PK–PD) models—can represent time-dependent changes in drug concentration (PK) and its physiological effects (PD) in the human body [30]. Therefore, these devices are advanced platforms for more precise prediction of drug efficacy, as well as their potential side effects in a monitored whole-body system compared to the single-organ models [16].

In particular, these multi-organ platforms can mimic metabolism-dependent drug actions, such as testing the efficacy of prodrugs undergoing chemical or enzymatic transformation to form active drug moieties with pharmacological effects [79,80]. For instance, human hepatic carcinoma cells and human mammary epithelial cells were cocultured on a microfluidic chip [62] to investigate amifostine prodrug metabolism and its resultant radioprotection effect on the living-tissue analog. The dual-tissue device was compared with a single-cell model, and a two-fold improvement in the effectiveness of the drug was observed in the system with the two cell types cocultured. In another study, the coculture of hepatic and lung cells on-chip enabled mimicking irinotecan prodrug hydrolysis in the liver and the consequent anticancer effect on the lung cells [29]. This was consistent with the bioactivation of this prodrug in the liver when administered orally in vivo [81].

By involving the interactions between multiple tissues, multi-organs-on-chips can enhance the physiological relevance and predictive capabilities of the simulated PK in vitro compared to mono-cultured organ models [50]. For instance, the first-pass metabolism of orally taken apigenin, a natural flavonoid, was modelled in a liver–gut-on-chip [28]. It was found that this system could improve the physiological relevance of the modelled metabolism process compared to the monoculture gut cells. The type of formed metabolites detected in the gut–liver device was similar to the first-pass metabolism of apigenin in a rat model. This was due to the contribution of both tissues in forming the different metabolites of apigenin.

Evaluating the responses from multiple organs to a novel therapy by multi-organs-on-chip platforms has shown to be promising at predicting the side effects and the off-target cytotoxicity responses to drugs. The undetected side effects of an approved drug put potential patients at risk of acute or chronic poisoning [82]. Therefore, evaluation of drug side effects at the early stages of drug discovery is of growing concern to protect public health and for the pharmaceutical industries, where the application of multi-organs-on-chips for drug testing in a physiologically representative microenvironment could be a potential solution. Promising results have been reported in the literature regarding the evaluation of drug side effects by multi-organs-on-chips [29,47,49]. Sung et al. reported the development of a microscale cell culture analog based on a mathematical PK–PD model with cocultured liver, tumor (colon cancer), and marrow cells to study the toxicity of an anticancer drug, 5-fluorouracil [52]. This device allowed the assessment of different responses to the test drug from each cell type consistent with clinical findings [83,84]. A multi-organs-on-chip system interconnecting liver spheroids with either intestinal epithelial cells or skin biopsies enabled the simulation of oral or systemic routes for troglitazone administration [48]. The simulation of oral drug exposure using this chip allowed biotransformation of the drug to its metabolites by the liver tissue, similar to in vivo results. Further, the simulation of systemic drug administration by this chip could mimic drug uptake by the fatty tissue underneath skin biopsies similar to in vivo. Another device, hosting four human organ analogs by coculturing the human small intestine, skin biopsy, liver, and kidney proximal tubule cells, was reported by Maschmeyer et al. [85]. These four organs-on-chip models enabled the in vitro reproduction of the oral and dermal drug absorption, first-pass hepatic metabolism, secondary metabolism, and renal metabolite excretion, emulating the in vivo absorption, distribution, metabolism, and excretion profiling of tested drugs and their toxicity.

Multi-organs-on-chip shed more insight into understanding drug action when treating human diseases. To demonstrate this, a study by Lee et al. developed a gut–liver chip to recreate hepatic steatosis, a process of abnormal lipid deposition in the liver cells [86]. The device emulated the anti-steatosis effects of butyrate, which acted on the gut tissue by enhancing its barrier function against the transport of free fatty acids. The mechanism underlying the influence of butyrate on the intestinal barrier function is known to be through promoting the assembly of TJs between the gut epithelial cells [87], and the use of this advanced platform enables understanding this.

#### 3.1.2. Simulation of Biomechanical Cues

**Figure 2:**
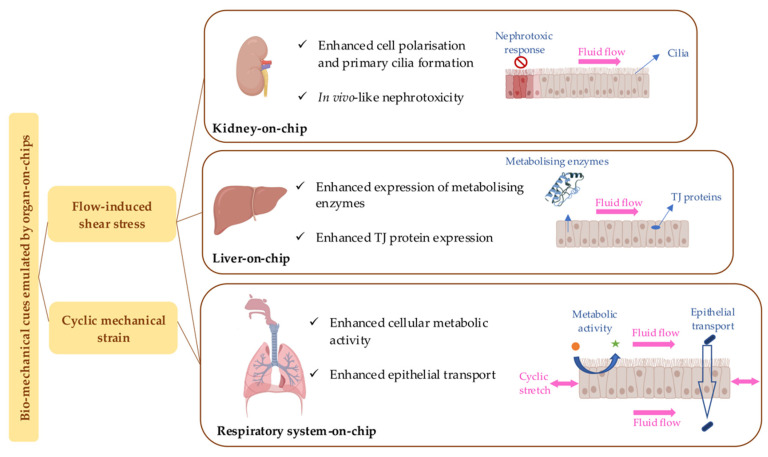
The biomechanical cues emulated by kidney-, liver-, and respiratory-system-on-chips and the summary of the observations in terms of the effects on the cellular morphology, permeability, and drug response. Figure is created with BioRender.com.

Numerous organ-on-chips have been designed to simulate mechanical stimuli present in vivo to recreate the in vivo-like dynamic microenvironment of tissues. In this regard, physiologically relevant physical cues such as motion, deformation, fluid flow, strain, and shear stress are simulated on-chip. Kidney-, liver-, and lung-on-chips have shown the dependence of the tissue’s physiological properties and functionalities on the mechanical cues. Figure 2 summarizes some of these effects. Hence, drug testing in organ-on-chips integrated with in vivo-inspired biomechanical factors is expected to deliver results closer to in vivo than conventional static cell cultures. As demonstrated, the flow-induced shear stress on primary kidney epithelial cells enhanced epithelial cell polarization and primary cilia formation in a human kidney proximal-tubule-on-chip [14]. Also, hepatic cells responded to the dynamic fluidic condition in a kidney–liver-on-chip by increasing the expression of the metabolism-associated biomarkers [65]. A lung-on-chip also showed that the stretching motion exerted on the primary human pulmonary alveolar epithelial cells could enhance cellular metabolic activity and biomarker secretion [13].

One of the important physiological characteristics of tissue-equivalence-on-chip models is the influence of mechanical stimuli on the tissue barrier function. For different tissue types cultured on organ-on-chips, it was reported that exposure to fluid flow could alter the paracellular permeability of tissue analogs on-chip compared to conventional static cultures [13,28,45,88,89,90]. Paracellular permeability refers to the passage of molecules through the intercellular spaces between adjacent epithelial cells. The tightness of small openings between the cells—the rate-limiting step in this process—is identified by the expression of TJ proteins. The fluid flow induces shear stress to the cells, which is reported to affect the expression of TJ proteins that consequently influence the paracellular permeability [45,88,90]. This is an important consideration when the permeation of drugs across the cell layer models are performed on organ-on-chip models where the obtained results could dictate the efficacy and toxicity of drugs [91,92]. For instance, the fluid-flow-induced shear stress on the liver and gut cells on-chip has been reported to accelerate cell differentiation, enhancing the epithelial barrier function and TJ protein expression, which, as a result, decreases the permeability of the cell layers modelled in these chips [28,31,45]. Another potential reason for the influence of flowing fluid on epithelial permeability is explained by the influence of the flow on the thickness of the unstirred water layer (UWL) [28]. UWL is an aqueous layer adjacent to the biologic solid–liquid interfaces that have a slow laminar flow and acts as an additional diffusion barrier against drug absorption [93,94,95]. The thickness of the UWL decreases with the fluid flowrate. This in turn increases the drug permeation when the diffusion through the UWL is the rate-limiting step [96,97].

In addition to the fluid flow, biomechanical strains are also found to influence the tissue barrier function in organ-on-chips. Mechanically active tissues—such as the lungs and GI tract tissues, which are exposed to stretches by breathing motions or peristalsis movements, respectively—are experiencing deformations and mechanical strains continuously [98]. A lung-on-chip model showed that the physiological strain on the lung epithelial cells resulted in an increase in the model’s permeability to hydrophilic molecules, while the cell layer’s integrity, TJs, and morphology remained unaffected [13]. This observation is in agreement with an in vivo investigation, where the increase in the lung volume by applying positive pressure to the airway influenced the permeation of hydrophilic solutes across the respiratory epithelium [99,100]. Further, simultaneous exposure of the gut epithelial cells to the fluid-flow shear stress and cyclic mechanical strain stimulated expression of a 3D villi-like morphology and an enhanced intestinal barrier function in a gut-on-chip [101]. Nevertheless, future studies are still required to elucidate the exact mechanism involved in the increase of permeability by mechanical strain at the molecular level.

Given the influence of biomechanical cues on the biological properties of the kidney, liver, and lung models, drug assays in such mechanically dynamic tissue models can potentially deliver different responses compared to the static models. Jang et al. compared the fluid dynamic cell-culture conditions in a kidney proximal-tubule-on-chip with the static culture in Transwell insert [14]. They found improved epithelial tissue morphology and kidney-specific functionalities under dynamic conditions. The dynamic model eventually showed closer in vivo human-relevant renal toxicity response to cisplatin treatment compared to the conventional static culture. The liver-on-chips with fluid-flow condition have also demonstrated increased expression of metabolizing enzymes and bioactivation of drug compounds through metabolism [26,33,36]. The enhanced metabolic functionality of these dynamic liver models can enhance their sensitivity to drug toxicity compared to the static cultures [33]. In addition, the exposure of the pulmonary epithelium to cyclic mechanical strain by a lung-on-chip was demonstrated to enhance cellular uptake and epithelial transport of the silica nanoparticles into the underlying microvascular channel [12]. This effect eventually accentuated both the toxic and inflammatory responses of the lung analog to silica nanoparticles, which is in agreement with in vivo observations.

#### 3.1.3. Drug Testing Using Modelled Human Diseases On-Chip

Another improvement in organ-on-chip technology that can increase the relevance of preclinical drug testing is its capability to recreate human disease on-chip. These are micro-structured models of human organs, where the organ analogs express key features of human diseases [63]. Diseased tissues are known to respond differently to drugs compared to healthy tissues [63]. In addition, animal models fail to mimic the complications shown in many human disease conditions [102]. Therefore, there is a need to develop disease-on-chips as more reliable models of human diseases to be used for the assessment of effective therapies.

The engineering of disease-on-chips includes novel platform designs to facilitate multiple analyses of human diseases in vitro. This enables understanding of the disease development mechanisms. For example, a study by Zhou et al. developed a liver-injury-on-chip integrated with biosensors for monitoring secreted transforming growth factor-β (TGF-β) triggered by alcohol injury [103]. Another feature of this is the reconfigurable structure that enabled the culture chambers of hepatocytes and stellate cells to be either isolated from each other or connected with the possibility for the two cell types to communicate. This feature enabled studies on the cellular origin of secreted TGF-β and found that alcohol injury causes hepatocytes to commence the secretion of TGF-β molecules, which activate the neighboring stellate cells and trigger additional TGF-β production by the stellate cells during development of alcoholic liver injury. Furthermore, disease-on-chips can pave the way to understand the role of organ–organ communication on the development and progression of diseases [78,104]. This was demonstrated in a study by Lee et al. that simulated hepatic steatosis using a gut–liver chip. The hepatic steatosis was induced on-chip by tumor necrosis factor-α, which increases the permeability of gut epithelium and, as a result, increases the level of lipid permeability to the liver tissue, leading to hepatic steatosis [86]. The knowledge and understanding gained on the mechanism of disease development from the organ-on-chip models will enable effective therapies to be developed.

The capability of organ-on-chips to mimic the dynamic microenvironments of human tissues is an asset to study human diseases, as these mechanically active platforms allow investigating the effect of physical cues on disease development [105] as well as the response of the diseased tissues to the test therapies [76,77]. The above can potentially help predict human response to new therapies with higher accuracy and develop novel therapies to treat human diseases effectively. This was demonstrated in a lung-on-chip model that aims to reproduce the drug-toxicity-induced pulmonary edema observed in human cancer patients treated with interleukin-2. The study showed that cyclic mechanical strain that is associated with the breathing motion in the lung tissue increases the likelihood of pulmonary edema development [105]. Based on this knowledge, a new inhibitor of the transient receptor potential vanilloid 4 ion channels (that are activated by mechanical strain) was tested as a potential preventive treatment. Interestingly, this therapy revealed satisfactory results in inhibition of pulmonary edema.

Taken together, disease-on-chips are reliable models and have shown promise for both understanding human diseases and developing novel effective therapies.

### 3.2. Integrated Sensing Tools in Organ-on-Chips for In Situ Drug Testing

Another advantage of organ-on-chips is their ability to integrate sensing tools in their designs, which enables in situ monitoring of the chip microenvironment. The sensors integrated into organ-on-chips can collect data from potential changes in the culture environment and cells’ biological properties in real-time. This can subsequently enhance the throughput of organ-on-chips used for drug tests [18]. The real-time monitoring of the cellular microenvironments by in situ analytical tools offers several advantages over the conventional analyses conducted off-chip, for example, shorter analysis time, cost-effectiveness due to reduced consumption of solvents, simpler operation, no requirement for sampling, elimination of the sampling errors, and lower risk of contamination, resulting in more reliable and accurate analyses. Several reviews have covered the application of biosensors in organ-on-chips for real-time monitoring of the physiological conditions of modelled tissue, focusing on cellular metabolism, function, and response to stimuli [106,107].

Recently, a liver-on-chip model was incorporated with commercialized amperometric glucose and lactate sensors that enabled the detection of minute shifts from oxidative phosphorylation to anaerobic glycolysis, indicating mitochondrial damage caused by drug concentrations previously considered as safe [19]. Furthermore, the integration of multiple sensors into the structure of organ-on-chips has paved the way for miniaturization and automation of various analyses in one platform without the need for large sample preparations [108]. Commercially available miniature microscopes, although challenging to incorporate in organ-on-chips at the moment, have been found helpful for monitoring the cells or cell-culture environment in real-time [35].

Another potential sensor that could benefit drug testing on organ-on-chips is the TEER measurement electrode. The integration of TEER measurement sensors in organ-on-chips has been reported for BBB-on-chip [109], heart-on-chip [110], pulmonary epithelium, and gut-on-chip models [109,110,111,112]. These devices, integrated with gold or platinum (Pt) electrodes, are reported to monitor the differentiation of cells and the formation of TJs in real-time. However, these chips have not been used in the context of drug testing to evaluate how drugs could affect TJ integrity and formation. However, they have the potential to facilitate the monitoring of TJ dynamics while treating the cells with novel drug formulations in terms of detection of toxicity effects of drugs on the cells or modulation of TJ dynamics by permeation-enhancing drug carriers [91].

Currently, there are still a very limited number of studies conducted to incorporate different sensors within organ-on-chips. Hence, there remains enormous potential for further development of versatile platforms that function like a living organ and are integrated with in situ sensing tools for real-time monitoring for pharmaceutical development. An example is the potential of using carbon-based electroanalytical sensors, which have proven to be suitable sensors for the electrochemical detection of numerous pharmaceuticals [113]. Since these sensors can offer reliable detection of drug compounds, integrating them with organ-on-chip designs can be useful in monitoring drug permeability and effects on-chip [73]. A human nasal epithelial mucosa-on-chip was developed—capable of real-time monitoring of both drug transport and TEER by housing the respective sensors in its structure [73]. This dual-channel chip was integrated with a carbonaceous electrode for in situ quantification of ibuprofen that was transported across the nasal epithelium to the acceptor channel. In addition, Pt electrodes were incorporated in the chip for in situ real-time TEER measurements. The presence of both sensors enables this chip to simultaneously measure the drug quantity in the cellular microenvironment and the potential variations in the barrier properties of the cell layer during drug testing. The in situ drug quantification by this chip was validated against high-performance liquid chromatography, which is the current gold standard for the quantification of pharmaceutics. In addition, the TEER measurements for the nasal epithelium model-on-chip were similar to what was reported for excised human nasal mucosa [114]. These features make this chip a potentially reliable alternative to the costly, time-consuming analytical techniques conventionally used for nasal drug assays.

### 3.3. Organ-on-Chips Enable Personalized Drug Testing

While integration of organ-on-chips with biological tissue or fluid specimens from patients, such as primary cells, biofluid samples, and cells derived from induced pluripotent stem cells, will enable emulation of patient-specific physiology, genetics, and biometric parameters on-chip [17,50], it also enables personalized organ-on-chips. These personalized chips can ultimately be used to stratify patients’ responses to drug exposure and develop personalized medicine or therapies. While it is challenging to test different drugs on a patient receiving therapies, a personalized organ-on-chip model developed for the specific patient could be of significant value in predicting the most efficient drug to treat their disease. The potential of this personalized model was demonstrated by Xu et al. who developed an in vivo-like tumor microenvironment using a microfluidic chip to assess anticancer drug efficacy on primary cells from fresh lung cancer tissues of eight patients to model individualized clinical treatment [115]. The cells were treated with different anticancer drugs: gefitinib, paclitaxel, and gemcitabine. This chip could mimic different drug sensitivities of the lung cancer tissues from different individuals. It also showed that the drug sensitivity of the patient-derived tissues was different from what was observed in conventionally used cell-line models. The comparison of drug responses from the patient-derived tissues on-chip and clinical trials on the patients should be the focus of future research in this area, helping to validate these devices in predicting clinical outcomes.

## 4. Disadvantages of Organ-on-Chips in Drug Testing

While there are numerous advantages for the use of organ-on-chips in facilitating drug testing and formulation-development processes, there are also several disadvantages. Polydimethylsiloxane (PDMS) is one of the main materials used for the fabrication of the majority of organ-on-chips. Despite the advantages, such as gas permeability and transparency, that make it a good option for cell culture and microscopic imaging purposes, it has been reported that PDMS can absorb some drug components with hydrophobic behavior [28,107]. This can adversely affect the outcomes of drug testing when hydrophobic compounds are investigated. However, this effect could be prevented by applying coatings on the surface of the PDMS [28].

Although the dynamic environment of organ-on-chips bridge the gap between the static tissue culture and the in vivo counterpart, it could also have potential disadvantages, where adverse effects involving the fluid flow on the cells have been reported, such as the detachment of the cells when exposed to high flow rates [28]. Jang et al. reported a negative influence of the perfusion flow on the matrix-embedded hepatocytes in a chip as they observed the premature death of the cells in the proximity of the perfusion channel. In contrast, the cells that were located distally from the perfusion channel were protected against this effect [33]. Zhao et al. reported that the high flowrate of the supplied medium into their microfluidic device washed the cells away because of the high shear force [116]. On the other hand, a low flowrate adversely affected the viability of the cells as it led to the shortage of nutrients in their chip to culture human non-small-cell lung cancer cells. In light of such observations, optimizing the microchannels’ dimensions and the extent of mechanical stimuli should be considered, as they appear to be important requisites to recapitulate physiologically relevant biomechanical cues. Such considerations may mitigate the potential negative effects of the dynamic microenvironments on the cells.

There is also currently no standardization of any of the organ-on-chip designs. Taking the lung tissue as an example, multiple organ-on-chip models with varying dimensions and architectures have been developed [12,13,64,117,118]. The differences in these models include presence [12,13] or absence [64,117,118] of mechanical strain, emulation of 2D [12] or 3D [13] breathing motions, and the type of cultured cells [12,13,117]. These differences can potentially result in different readouts from each of these models for a similar assay. Further, the variables inherent with the different models will not be useful for a highly regulated setting, such as the pharmaceutical industry, where reproducibility and standardization are essential. Consequently, it may not be suitable to compare the findings from different organ-on-chip designs, which highlights the need for the introduction of a standardization system for all organ-on-chip models that recreate the physiology of the same tissue. This highlights the necessity of defining standards for design and operation factors considered in development of organ-on-chip models for a certain organ.

## 5. Conclusions and Future Outlook

Organ-on-chips are found to be promising alternatives to the conventional cell culture models for enhancing the predictive power of preclinical drug tests. These platforms enable monitoring drug metabolism pathways, toxicity effects on single or multiple connected organs, and drug delivery to a target organ while also improving the time efficacy and reliability of the readouts.

Despite the ongoing progress in the design and development of biomimetic organ-on-chips in a plethora of research studies, this technology has yet to accomplish its ultimate level of advancement: mimicking the complex physiology of the whole human body to be potentially accepted as an alternative to animal models or preclinical trials. Although multi-organs-on-chips are widely studied to recreate human response to numerous drugs, they are far from a full human body-on-chip, with still many organs to be explored. Future research can focus on incorporating several organs in multi-organs-on-chips combined with coculture models that will significantly enhance the accuracy of evaluating drug actions and side effects in the whole human body.

In addition, further work should focus on introducing standardized design factors, such as the ratio of dimensions, fluid volume, flowrate, shear stress, and biomechanical forces, for the development of organ-on-chips which can be used in highly regulated settings (e.g., the pharmaceutical industry). This will help ensure that every novel organ-on-chip design can mimic the physiological features of the tissue according to stated standards. These factors can also be presented in the form of nondimensional numbers in order to reduce the number of parameters needed to categorize the key physical and mechanical attributes of a particular device, as is done in the engineering of drug-delivery devices [119,120,121]. Hence, researchers could compare the results from different models of one tissue type with the obtained experimental data from multiple studies being used as a valid reference for potential future clinical studies with discrepancies less-likely.

While organ-on-chip models to study the functionality of organs and disease states have progressed significantly over the last decade, they rely on off-chip analysis and imaging techniques, which are labor-intensive and time- and cost-consuming. Integration of continuous, non-invasive, in-site, real-time monitoring of the functionality of the tissues is in significant demand to address this problem. While current efforts are focused on the physical and biochemical cues of the physiological microenvironment, such as cell-secreted molecules and TJ integrity, there remains a significant underexplored area of using these microfluidic platforms equipped with drug sensors for preclinical drug testing in a physiologically relevant environment. This can potentially accelerate the translation of drugs to clinic, where organ-on-chips can play a significant role in the development pipelines in future drug discoveries as more accurate and reliable alternatives to conventional in vitro analyses or animal trials.

## Figures and Tables

**Table 1:** Overview of the primary focus of relevant reviews in the application of OOCs for preclinical drug testing compared with the current review.

Refs.	Focus of the Review
[3,4,5]	Application of organ-on-chip models of certain organs in providing a relevant platform for drug testing in terms of the design, structure, and cell culture techniques The different assays used to evaluate drug efficacy in those organ-on-chip models
[3,9,10,11]	Progress, challenges, and opportunities for the application of organ-on-chip technology in preclinical drug discovery The commercialization outlook of organ-on-chip technology for drug testing
[6,7]	Drug toxicity studies performed on organ-on-chips with improved physiological relevance
[8]	Organ-on-chips as potential platforms for screening nanocarrier drug delivery with improved physiological relevance
This review	Improvements of in vitro drug efficacy assays when conducted in organ-on-chips to ensure outcomes are clinically relevant Advantages of organ-on-chip technology in providing a translational model for physiologically relevant in vitro drug testing Discussion of the various drug compounds that have been tested on organ-on-chips Relevance of drug testing outcomes from organ-on-chips to clinical observations
